# The Sonographic Muscle Injury Classification in Soccer: proposal for an ultrasound-based muscle injury classification

**DOI:** 10.1590/0100-3984.2025.0112

**Published:** 2026-09-27

**Authors:** Roberto Mogami, Claudio Henrique Ivo de Araújo Ribeiro, Eduardo Brown Guedes dos Santos, André de Almeida Vieria, Lucas Nascimento da Luz, Caio Leal Leidersnaider, Gabriela de Oliveira Sarmento, Paulo Sérgio Chagas Gomes

**Affiliations:** 1 Department of Diagnostic Imaging, Universidade do Estado do Rio de Janeiro, Rio de Janeiro, RJ, Brazil; 2 Vasco da Gama SAF, Rio de Janeiro, RJ, Brazil; 3 Hospital Universitário Pedro Ernesto, Universidade do Estado do Rio de Janeiro, Rio de Janeiro, RJ, Brazil; 4 Department of Radiology, Universidade Federal do Rio de Janeiro, Rio de Janeiro, RJ, Brazil; 5 Department of Radiology, Suprema Faculdade de Ciências Médicas de Três Rios, Três Rios, RJ, Brazil; 6 Department of Exercise and Sport Sciences, Universidade do Estado Rio de Janeiro, Rio de Janeiro, RJ, Brazil

**Keywords:** Ultrasonography, Muscle, Soccer, Classification, Magnetic resonance imaging, Ultrassonografia, Músculos, Futebol, Classificação, Imageamento por ressonância magnética

## Abstract

**Objective:**

To propose a novel ultrasound-based classification system for muscle injuries in soccer players, termed the Sonographic Muscle Injury Classification in Soccer (SOMICS), and to evaluate its association, ordinal consistency, and agreement with the British Athletics Muscle Injury Classification (BAMIC).

**Materials and Methods:**

This was a cross-sectional observational study including 51 adolescent male soccer players (11–19 years of age) with suspected acute myotendinous injuries. All athletes underwent ultrasound and magnetic resonance imaging (MRI) within 24 h after injury. Four experienced radiologists, working independently, reviewed the images.

**Results:**

Interobserver agreement was substantial for ultrasound (κ = 0.65) and MRI (κ = 0.70). A strong ordinal correlation was observed between SOMICS and BAMIC categories (ρ = 0.80; *p* < 0.001), with a statistically significant association (χ^2^ = 52.53; *p* < 0.001).

**Conclusion:**

The SOMICS demonstrated significant association, ordinal consistency, and substantial agreement with the BAMIC for severity stratification of muscle injuries. Although not intended to replace MRI-based classifications for prognostic decision-making, the SOMICS provides a structured, reproducible ultrasound-based approach that may be useful in clinical settings where MRI is not readily available or as a first-line imaging tool.

## Abstract (pt)

**Objetivo:**

Propor uma nova classificação ultrassonográfica para lesões musculares em jogadores de futebol, denominada SOMICS (Sonographic Muscle Injury Classification in Soccer), e avaliar sua associação, consistência ordinal e concordância com a British Athletics Muscle Injury Classification (BAMIC).

**Materiais e Métodos:**

Estudo observacional transversal que incluiu 51 atletas do sexo masculino, adolescentes (11–19 anos), com suspeita de lesões miotendíneas agudas. Todos os atletas foram submetidos à ultrassonografia (US) e à ressonância magnética (RM) em até 24 horas após a lesão. Quatro radiologistas experientes analisaram independentemente os exames.

**Resultados:**

A concordância interobservador foi substancial tanto para a ultrassonografia (kappa = 0,65) quanto para a ressonância magnética (kappa = 0,70). Observou-se forte correlação ordinal entre as categorias SOMICS e BAMIC (rho de Spearman = 0,80; *p* < 0,001), com associação estatisticamente significativa (qui-quadrado = 52,53; *p* < 0,001).

**Conclusão:**

A SOMICS demonstrou associação significativa, consistência ordinal e concordância substancial com a BAMIC na estratificação da gravidade das lesões musculares. Embora não tenha como objetivo substituir as classificações baseadas em ressonância magnética para tomada de decisões prognósticas, a SOMICS oferece uma abordagem ultrassonográfica estruturada e reprodutível, que pode ser particularmente útil em cenários clínicos nos quais a RM não esteja prontamente disponível ou como método de imagem de primeira linha.

## INTRODUCTION

Muscle injuries account for 20–37% of all sports-related injuries in men and for 18–23% of those in women^**(^1^–^4^)**^. Ultrasound and magnetic resonance imaging (MRI) are the primary diagnostic modalities for estimating recovery time and reducing the risk of recurrence^**(^1^–^3^,^5^–^7^)**^. In recent years, multiple studies and consensus statements have aimed to standardize the assessment of muscle injury severity^**(^8^,^9^)**^. A prominent classification system, the British Athletics Muscle Injury Classification (BAMIC), is based on MRI findings of hamstring lesions and categorizes injuries into five grades, from 0 to 4^**(^10^)**^. According to the anatomical location of the lesion, BAMIC grade 1 is subdivided into sections A and B, whereas BAMIC grades 2 and 3 are subdivided into sections A, B, and C^**(^10^)**^.

Although ultrasound is widely used to diagnose muscle injuries, its role in systematically grading injury severity remains limited. The classification devised by Peetron^**(^11^)**^, the most frequently cited ultrasound-based classification, provides a straightforward framework but lacks detailed anatomical stratification, excludes vascular assessment, and offers limited ability to estimate injury severity or predict prognosis. Another ultrasound-based classification, devised by Pedret et al.^**(^12^)**^, is aimed at improving the severity assessment by targeting the medial head of the gastrocnemius and focusing on lesion location and extent. Despite representing progress, this method is restricted to a single muscle and does not offer a comprehensive framework for multiple muscle groups or diverse injury mechanisms. Therefore, there is still a need for a general, standardized, anatomy-oriented ultrasound classification that integrates structural damage with biological activity and allows correlation with established MRI-based systems such as the Munich Consensus Classification^**(^13^)**^ or the BAMIC^**(^10^)**^. To address this need, as a primary objective of this study, we propose the Sonographic Muscle Injury Classification in Soccer (SOMICS) as a new ultrasound classification system, using the BAMIC as a reference for stratifying ultrasound severity. Secondary objectives were to evaluate whether a significant association, ordinal consistency, and categorical agreement exist between the SOMICS and BAMIC.

## MATERIALS AND METHODS

### Study design

This was an cross-sectional observational study including athletes recruited from the football (soccer) club Vasco da Gama SAF, conducted as an exploratory and developmental investigation between June 2022 and October 2024. The project was approved by the Research Ethics Committee of Hospital Universitário Pedro Ernesto (Reference no. 56309422.7.0000.5259), located in the city of Rio de Janeiro, Brazil.

### Sample

A convenience sample was used in order to select consecutive athletes suspected of having acute myotendinous injuries during matches, training sessions, or gym workouts. These athletes were referred for MRI and ultrasound examinations within the first 24 h after the injury occurred.

### Inclusion criteria

Male youth soccer players 11–19 years of age who were enrolled in the soccer academy of a competitive club were eligible for inclusion. Eligibility required presenting with acute onset of pain during soccer activity, consistent with an indirect (non-contact) mechanism of muscle injury, irrespective of anatomical location. Clinical findings necessary for inclusion comprised localized pain, tenderness on palpation, pain during active contraction or passive stretching, and functional limitation.

### Exclusion criteria

Individuals with a history of direct trauma, previous surgery involving the affected limb, or known chronic muscular disorders were excluded, as were those in whom the imaging evaluation was incomplete and those diagnosed with non-muscular conditions on MRI, such as apophyseal avulsion fractures, apophysitis, or bone stress injuries. We also excluded those who were using medications that could significantly alter muscle healing or imaging findings at the time of injury.

This study served as an exploratory validation of an ultrasound-based classification system in a youth population. To our knowledge, there are as yet no adolescent-specific muscle injury classifications. Therefore, the BAMIC, originally developed for adult athletes, was selected for comparison.

The weighting of the SOMICS components was defined *a priori*, drawing on cumulative clinical and imaging experience in sports medicine and musculoskeletal radiology, particularly regarding the prognostic relevance of anatomical involvement, tissue disruption, and vascular response. Subsequent statistical analyses were conducted to evaluate its association, ordinal coherence, and agreement with the BAMIC.

### Ultrasound and MRI readers

All readers had a minimum of 10 years of musculoskeletal imaging experience. Prior to image interpretation, a calibration session was conducted using representative cases to standardize grading criteria. For intraobserver analysis, readings were repeated after an interval of four weeks to minimize recall bias. The ultrasound scans were interpreted by two radiologists, working independently: one with over 20 years of experience in musculoskeletal ultrasound; and the other with over 10 years of experience in the field. Neither had access to the MRI results, although both had access to the clinical data. The MRI examinations were interpreted by the same two radiologists, blinded to all other results except for the clinical and sports data.

### Ultrasound equipment

The examinations were conducted with a Logiq 10 ultrasound system (GE Healthcare, Chicago, IL, USA); 14 MHz linear and 20 MHz “hockey stick” transducers were used. Images were obtained in B-mode and power Doppler studies, with a pulse repetition frequency set at 750 Hz, a low-wall filter, and gain adjusted to remain below the image saturation level.

### Ultrasound protocols

The scans were performed in the longitudinal and transverse planes, with constant comparison with the contralateral side, at rest and during isometric contraction maneuvers. The anterior thigh was evaluated from the iliac crest to the distal insertion of the quadriceps tendon. The posterior thigh was evaluated from the ischial tuberosity to the popliteal fossa. The region between the pubis and the distal femur was examined to assess the adductor muscles. The calf was evaluated from the popliteal fossa to the insertion of the Achilles tendon. Power Doppler was applied to the scan areas to detect and quantify anomalous vascular flow.

### Ultrasound terminology

Increased muscle echogenicity was characterized by loss of fascicular and fibroadipose septal definition (in the perimysium). According to Paoletta et al.^**(^4^)**^, this change results from increased capillary permeability and rupture of small capillaries, which can generate microhemorrhages in adjacent tissues. The erythrocytes from these hemorrhages act as small reflectors, conferring hyperechogenicity to the muscle on ultrasound (Figure 1).

**Figure 1:**
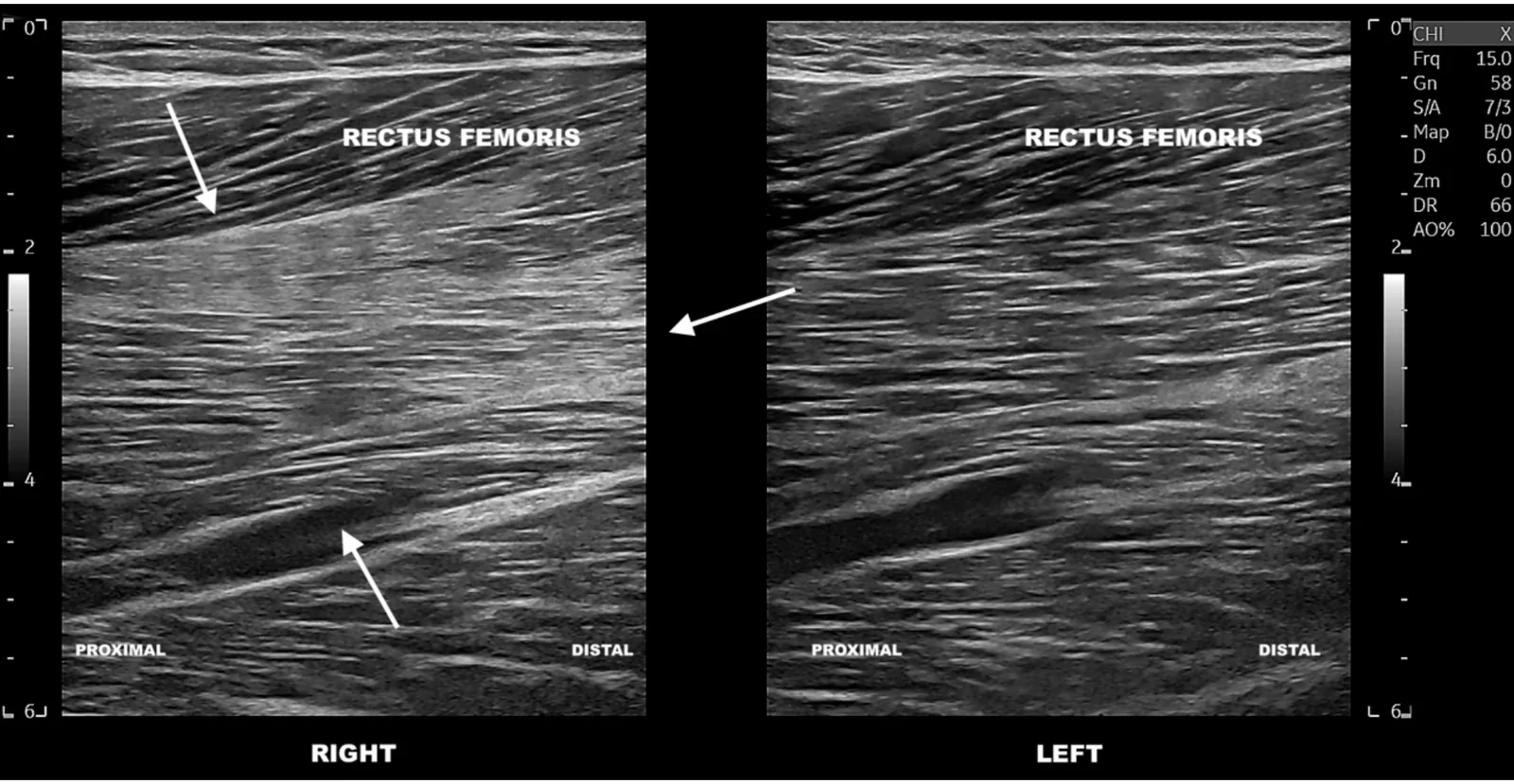
A 17-year-old male with sudden pain in the right thigh while playing a match. Comparative ultrasound images of the anterior region of the rectus femoris in the sagittal plane. The right side shows increased echogenicity and a lack of definition of the normal fibroadipose septa and fascicle pattern due to edema (arrows). SOMICS category 1 (score of 4).

Discontinuity of fascicles in at least two planes of study characterized muscle and tendon tears (Figure 2). A complete tear was defined as one affecting the entire thickness of the examined segment, often resulting in retraction of the ends. In contrast, a partial tear only involved a portion of the thickness. When the alteration was restricted to the fascicles and perimysium, without tendon or epimysial involvement (Figure 1), the involvement was classified as muscular. Myofascial/aponeurotic involvement was defined by thickening or discontinuity of the aponeurosis and the presence of fluid in the epimysial space (between aponeuroses), as illustrated in Figure 3.

**Figure 2:**
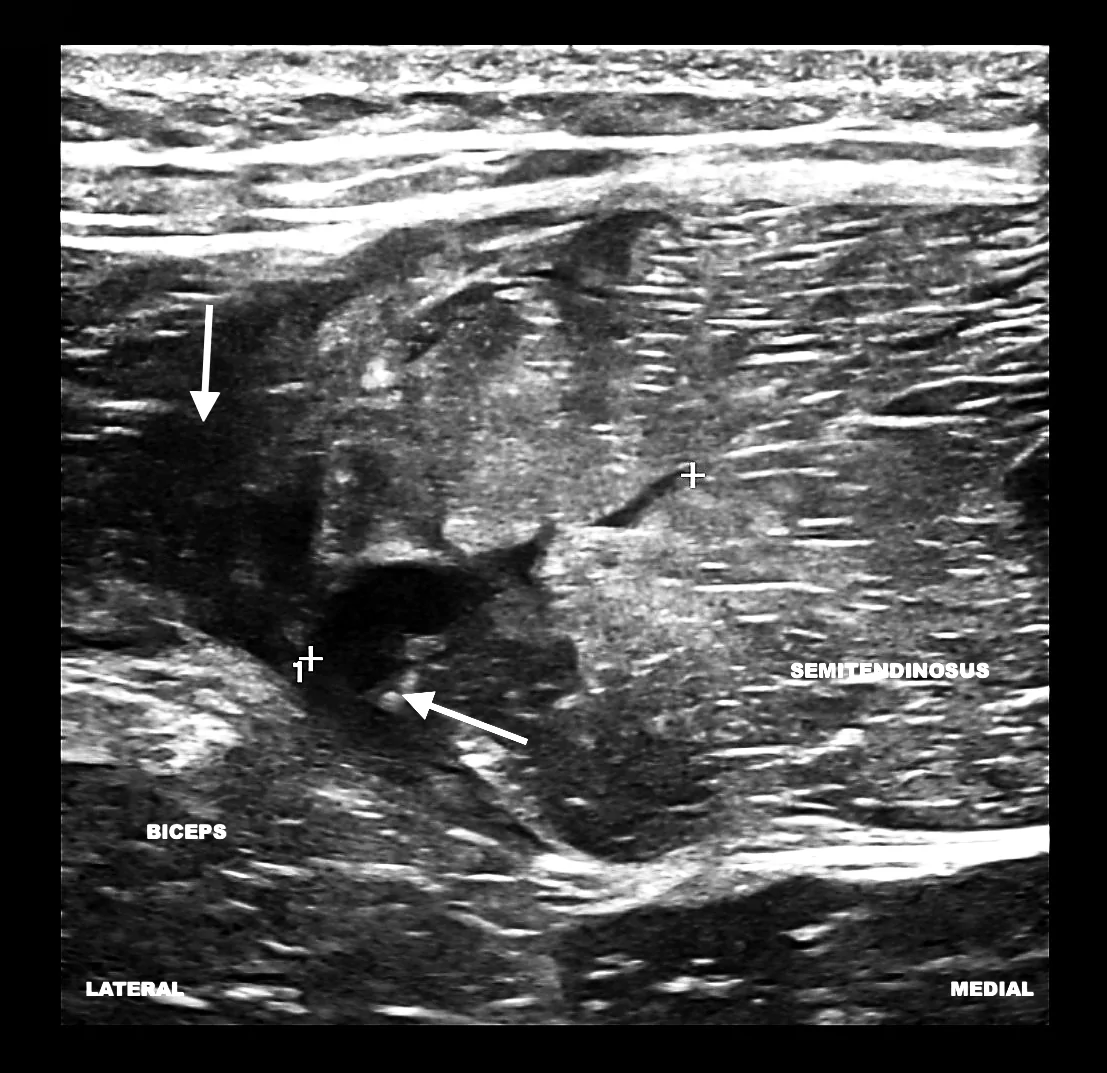
A 15-year-old male felt a sudden “pop” after stretching his leg to reach the ball while training. An ultrasound image in the transverse plane of the semitendinosus muscle showing a partial tear of the muscle fascicles and a complete tear of the conjoined tendon, where there was an anechoic collection (arrows). SOMICS category 3 (score of 11).

**Figure 3:**
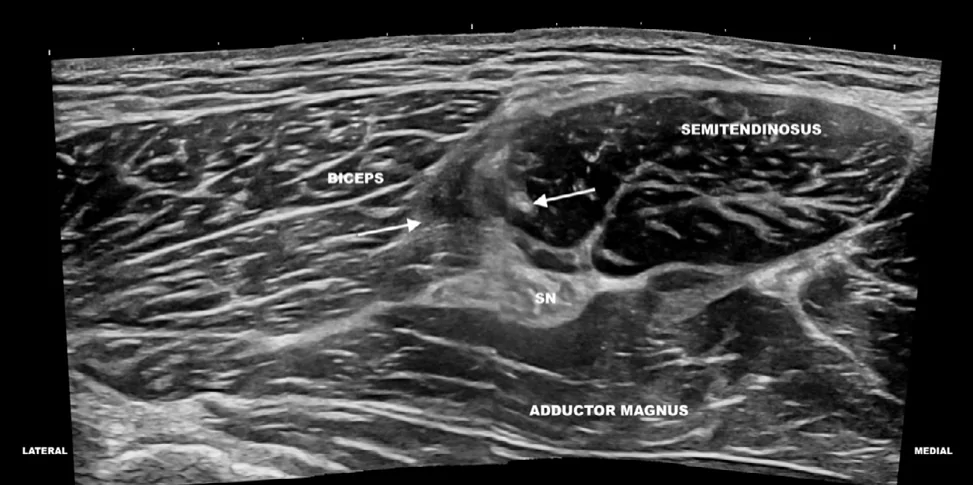
A 17-year-old male complained of a posterior soreness in the thigh after a match. An ultrasound image in the transverse plane of the semitendinosus and biceps muscles showing architectural distortion of both muscle fascicles, disruption of the conjoined tendon (arrows), and a partial tear of the semitendinosus aponeurosis. SOMICS category 3 (score of 8). SN, sciatic nerve.

A thickened, tortuous, or discontinuous tendon characterized tendinous involvement. Myotendinous involvement was characterized by alterations in the transition between the muscle fascicles and the tendon (Figures 4 and 5). The percentage of the cross-sectional area injured was estimated at the level of the most extensive lesion.

**Figure 4:**
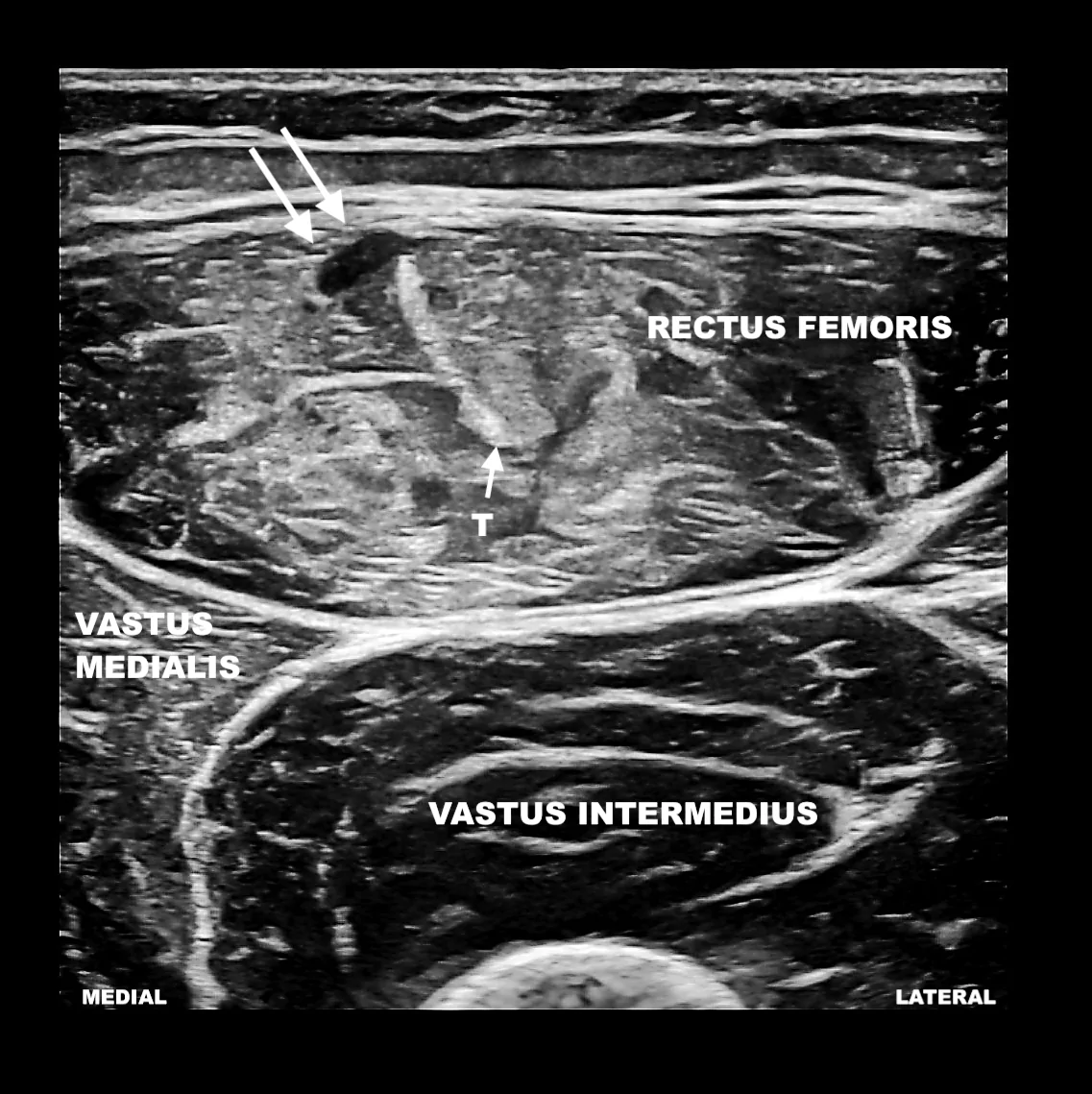
A 13-year-old male with sudden pain in the anterior region of the left thigh after kicking a ball. An ultrasound image in the transverse plane of the rectus femoris showing a myotendinous injury with edema and architectural distortion of the fascicles around the tendon (single arrow). Note the hypoechoic focal lesion (double arrows) next to the tendon, due to a muscle injury. SOMICS category 2 (score of 7). RF, rectus femoris; T, tendon.

**Figure 5:**
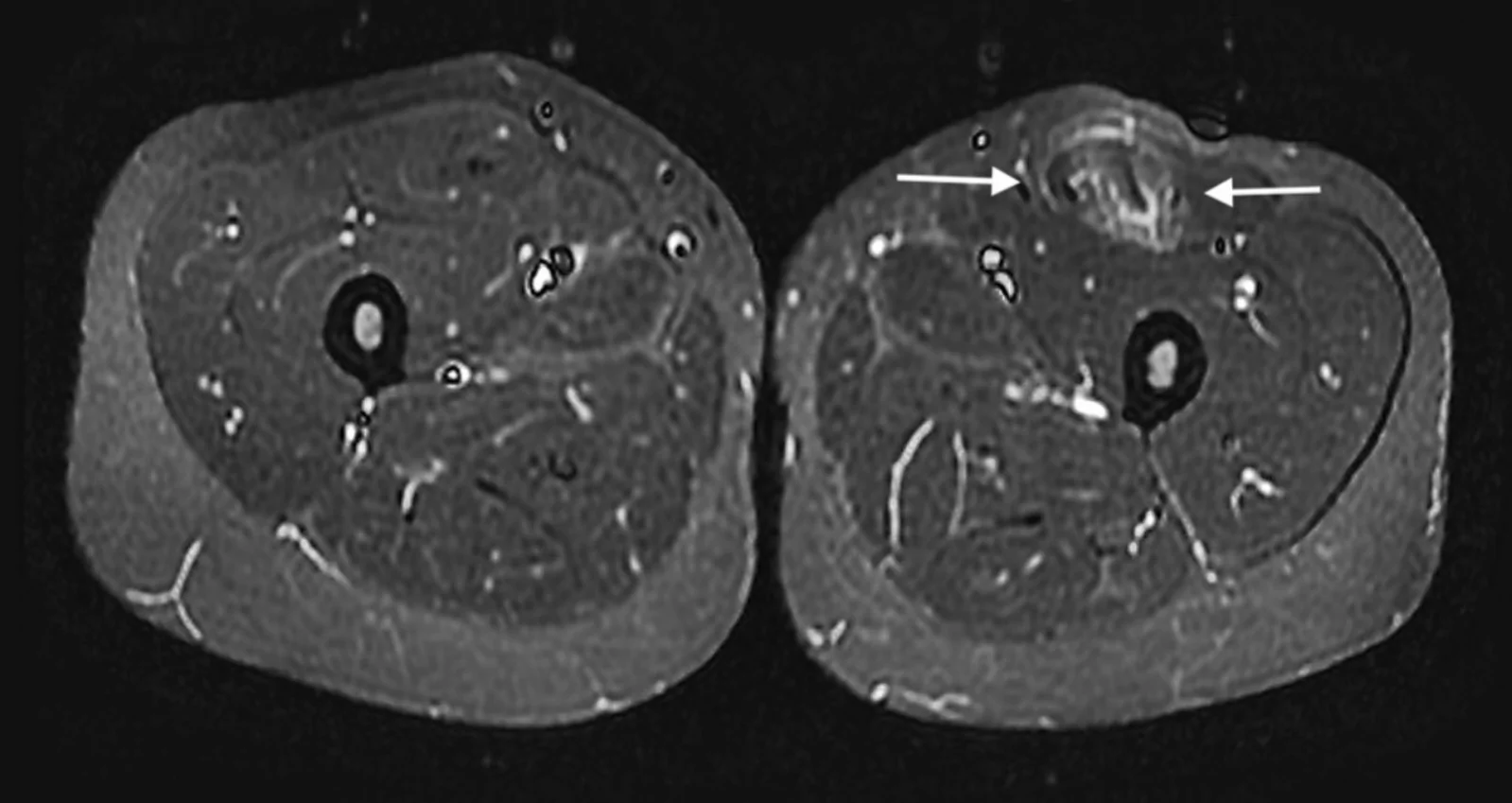
A 13-year-old male with sudden pain in the anterior region of the left thigh after kicking the ball. Axial short-tau inversion recovery MRI sequence of the thigh showing edema and architectural distortion of the myotendinous junction of the indirect rectus femoris tendon (arrows). SOMICS category 2 (score of 7).

Power Doppler (Figure 6) was used in order to identify increased flow that was asymmetric relative to the contralateral side. The intensity of the anomalous flow was graded according to the number of flow signals, as follows: mild (1–3 flow signals); or intense (> 3 flow signals).

**Figure 6:**
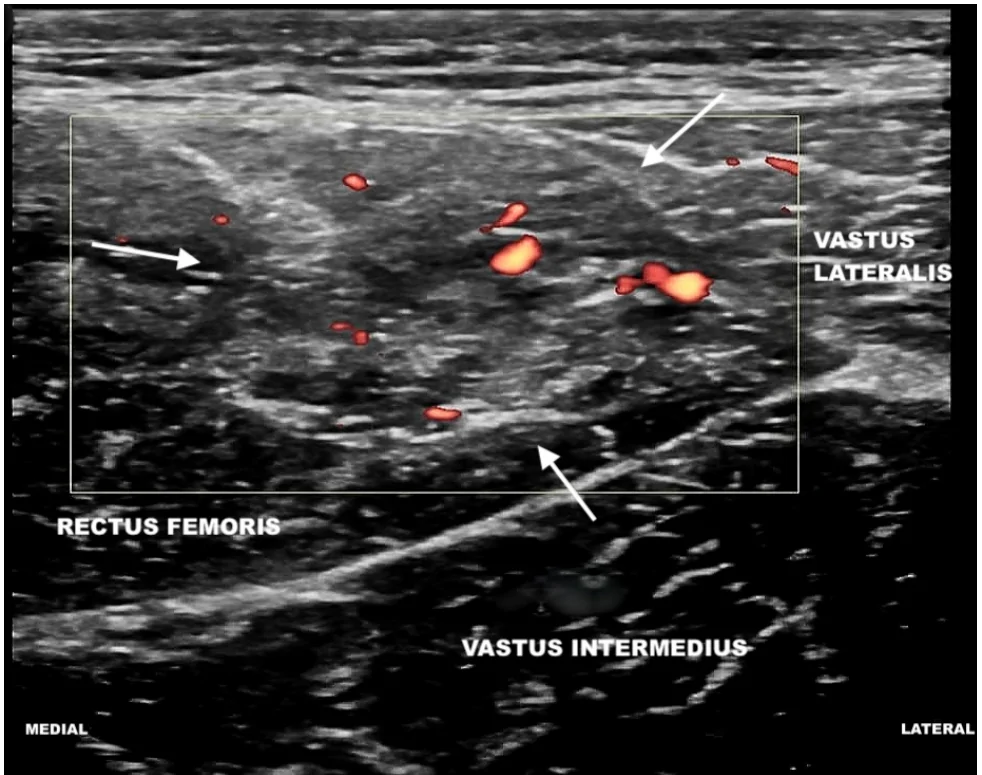
A 13-year-old male with sudden pain in the anterior region of the left thigh after kicking the ball. Image in the transverse plane of the rectus femoris showing architectural distortion of the fascicles on grayscale ultrasound. Vascular hyperflow on power Doppler (arrows) may indicate the formation of reparative tissue. There are more than three flow signals in this image. SOMICS category 2 (score of 7).

### SOMICS scores and statistical analysis

The first section of the SOMICS assesses the degree of anatomical impairment. Thus, anatomical impairment was divided into three situations (Table 1): mild injury with interstitial hemorrhage; a partial tear; and a complete tear.

The second SOMICS section considers anatomical location as a determining factor in injury severity. Tendon injuries receive the highest scores (Table 1).

The third section was included on the basis of the experience of the authors in sports medicine and the conclusion that the involvement of a greater number of muscles results in greater functional and anatomical impact (Table 1).

**Table 1:** SOMICS scores.

Degree of anatomical impairment	Points
Increased muscle echogenicity or intermuscular hematomas, without fascicle tears	1
Fascicle tear < 10 mm	2
Fascicle tear of 10–50 mm	3
Fascicle tear > 50 mm	4
Complete tear	5
**Location**	
Muscular	1
Myofascial/myoaponeurotic	2
Myotendinous junction	3
Tendinous	4
**Number of muscles affected**	
1	1
2–3	2
> 3	3
**Proportion of cross-sectional area altered**	
< 25%	1
25–50%	2
> 50%	3
**Power Doppler**	
1–3 flow signals	1
> 3 flow signals	2

The fourth section adapts the BAMIC descriptions regarding the anatomical extent of the injury. However, because longitudinal measurement using extended reconstruction may not be as accurate as that obtained with MRI, only the percentage of cross-sectional area involved at the most affected level was considered (Table 1). To estimate the extent of injury, the axial image showing the greatest tissue involvement was first identified. Electronic calipers were used in order to manually delineate the perimeter of the injured area and the total anatomical cross-sectional area of the muscle or myotendinous unit in the same image. The ultrasound system software automatically calculated both measurements. The injury percentage was determined by dividing the injured area by the total cross-sectional area at the corresponding level (Figure 5).

The fifth section incorporates power Doppler assessment to evaluate abnormal vascular flow within the injured area. As no previously validated Doppler-based grading system exists for muscle or tendon tears, this component was adapted from established rheumatologic ultrasound scoring systems for synovitis. Similar to those classifications, vascularity was graded according to the number of Doppler flow signals detected within the lesion, allowing stratification of inflammatory and reparative activity^**(^14^)**^, as detailed in Table 1.

The statistical analysis aimed to validate the proposed classification by assessing its association, correlation, and agreement with the reference (the MRI-based BAMIC). Ultrasound scores were first grouped according to the four BAMIC severity grades. Descriptive statistics (means and medians) revealed a progressive increase in SOMICS scores across the BAMIC grades, confirming ordinal coherence.

To determine the optimal boundaries between SOMICS categories (0, 1, 2 and 3), two complementary approaches were used. The first was **distributional**, in which midpoints between adjacent medians defined the score intervals corresponding to increasing injury severity. The second was **inferential**, based on **cutoff optimization**: each possible ultrasound score was tested as a threshold between the BAMIC grades by calculating its sensitivity and specificity. The optimal cutoff point was identified by using **Youden’s index** (sensitivity + specificity − 1), which provided the best balance between true-positive and false-positive rates.

The **strength of association** between SOMICS categories and BAMIC grades was evaluated using **Pearson’s chi-square test** and **Cramer’s V coefficient**. **Spearman’s rho** was calculated to quantify the correlation between continuous ultrasound scores and the BAMIC grades, confirming the ordinal trend between the two scales. Finally, **quadratic weighted Cohen’s kappa** and **overall accuracy** were calculated to quantify categorical agreement and the consistency of severity classification between the SOMICS and the BAMIC.

**Figure 7:**
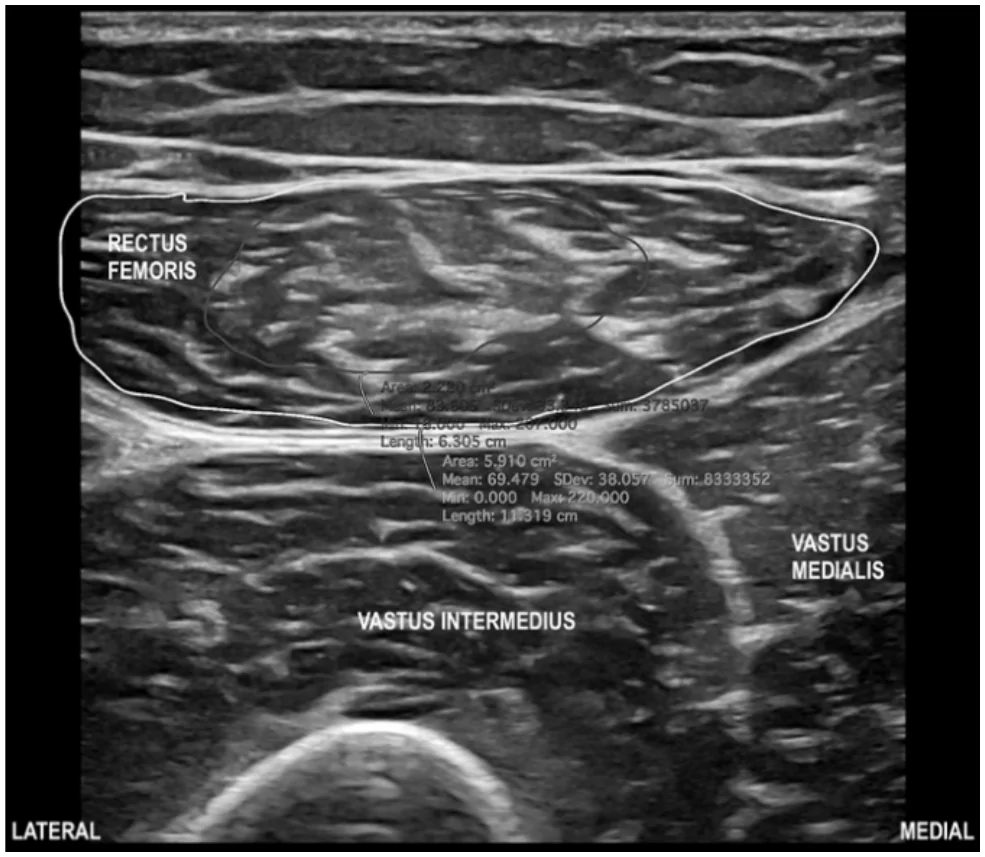
Measurement of the cross-sectional area of an injury. A 15-year-old athlete presented with anterior right thigh pain after sprinting to catch a ball. A transverse ultrasound image of the rectus femoris showing measurement of the central injured area (2.22 cm^2^) and the total cross-sectional area of the rectus femoris at this level (5.91 cm^2^). The alteration affected 37.6% of the total muscle area.

**Figure 8:**
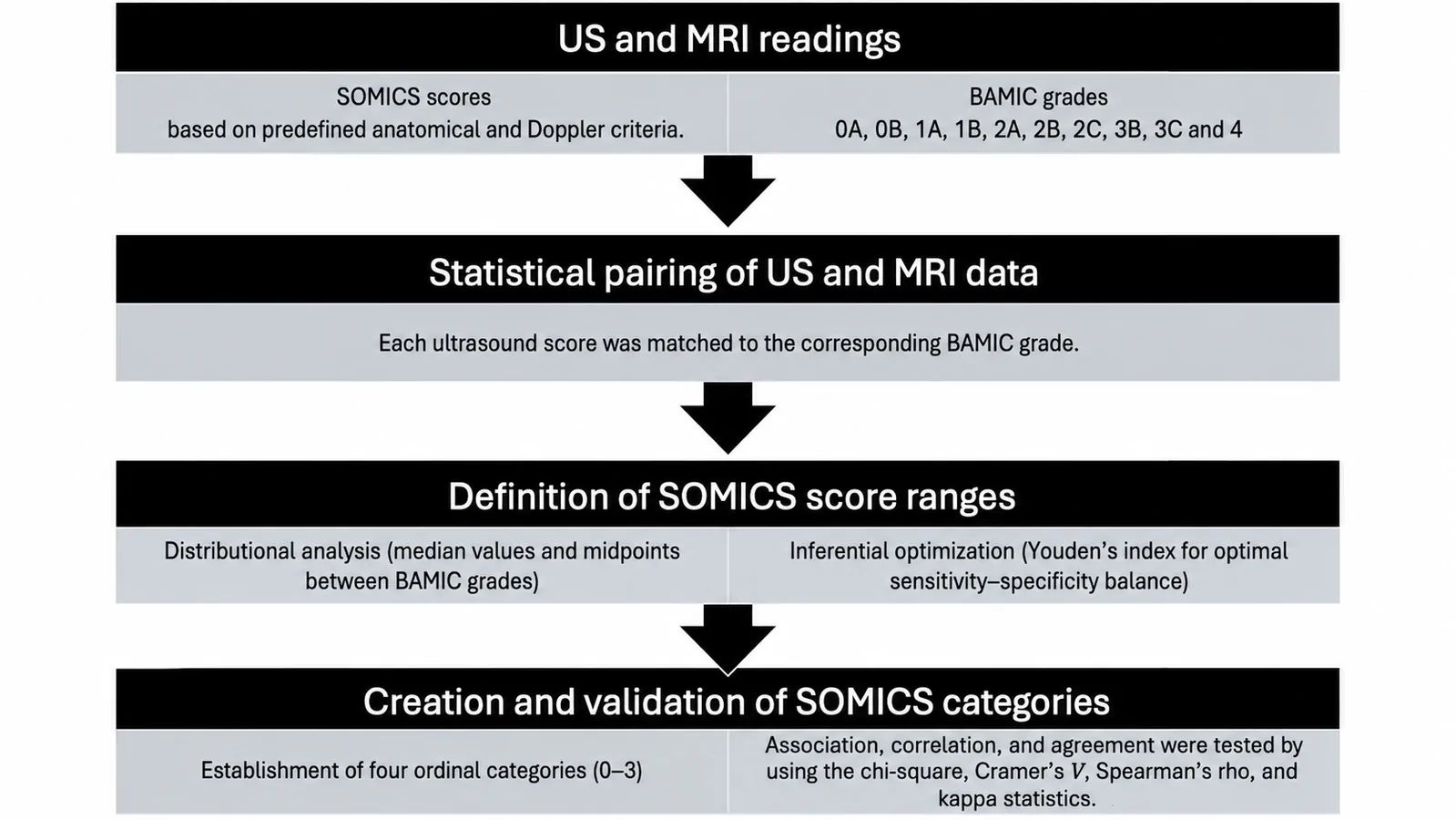
Sequential stages to arrive at the SOMICS categories.

All analyses were performed with the IBM SPSS Statistics software package, **version 26.0 (IBM Corp., Armonk, NY, USA)**, with a significance level of **p < 0.05**. The diagram in Figure 6 illustrates the sequential stages involved in determining the SOMICS categories.

## MRI

In determining the ultrasound severity categories, MRI was used as a reference. The MRI examinations were performed in a 1.5-T scanner (Optima MR 360; GE Healthcare), using T1-weighted and short-tau inversion recovery sequences in the axial and coronal planes, without intravenous contrast administration.

The severity of muscle injuries was assessed by using the BAMIC as a reference. Due to sample size constraints and statistical robustness requirements, certain BAMIC subgrades were aggregated for analytical purposes. This decision was methodological rather than conceptual, and future external validation studies should aim to preserve full tendinous stratification.

## RESULTS

### Descriptive analysis

The sample consisted of 51 male athletes, with a mean age of 15.8 years (range, 11–19 years). Of the 51 athletes evaluated, 47 (92.2%) played field soccer and four (7.8%) played field and indoor soccer. Two (3.9%) were goalkeepers, eight (15.7%) were defenders, 10 (19.6%) were full-backs, 15 (29.4%) were midfielders, and 16 (31.4%) were forwards (Supplementary Table 1). Suspected injuries occurred after matches (in 33.3%), after field training (in 60.8%), or after strength training (in 3.9%). Recurrent injuries occurred in 11 cases (21.6%).

Of the 51 ultrasound examinations 27 (52.9%) were normal, compared with only 17 (33.3%) of the MRI examinations. On MRI, the following muscle groups were affected: hamstrings, in 13 (25.5%); quadriceps, in eight (15.7%); adductors, in six (11.8%), obturator externus, in three (5.9%); iliopsoas, in two (3.9%); and gastrocnemius, in one (2.0%).

### Inferential analysis

Intraobserver and interobserver agreement in ultrasound and MRI readings for diagnosing myotendinous injuries were evaluated (Supplementary Table 2). For ultrasound, intraobserver analyses revealed simple agreement of 96.1% (κ = 0.92; 95% CI: 0.80–1.00) and 94.1% (κ = 0.86; 95% CI: 0.67–1.00) for readers 1 and 2, respectively, an excellent level of agreement according to the Landis and Koch criteria. In the interobserver evaluation, simple agreement of 86.3% (κ = 0.65; 95% CI: 0.43–0.84) was observed, corresponding to substantial agreement. For MRI, intraobserver analyses revealed simple agreement of 94.1% (κ = 0.86; 95% CI: 0.68–1.00) and 98.0% (κ = 0.96; 95% CI: 0.87–1.00) for readers 1 and 2, respectively, indicating excellent reproducibility. The interobserver analysis between the two readers showed a simple agreement of 86.3% (κ = 0.70; 95% CI: 0.49–0.88), a substantial level of agreement according to the Landis and Koch criteria.

As a reference for the development of the SOMICS, we employed the BAMIC, which categorizes injuries into four severity grades (0A/0B, 1A/1B, 2A/2B/2C, and 3B/3C/4) based on distributional and statistical criteria. The ultrasound (SOMICS) scores were distributed as follows (Supplementary Table 3): 27 cases with a score of 0, 12 with a score of 1, four with a score of 2, 11 with a score of 3, five with a score of 4, seven with a score of 5, eight with a score of 6, and two with a score of 7. Supplementary Table 4 shows the BAMIC results. The BAMIC grade assigned was 0A in 17 cases, 0B in two, 1A in five, 1B in nine, 2A in one, 2B in four, 2C in three, 3B in three, 3C in five, and 4 in two.

### Distributional criteria

The SOMICS scores were grouped according to the four BAMIC grades. For each group, the means and medians of the scores were calculated, revealing a monotonic progression pattern with medians of 0, 5, 6, and 10 points, respectively. The boundaries between ranges were defined by the midpoints between adjacent medians, resulting in four progressive intervals of severity, representing distinct stages of impairment while maintaining ordinal consistency (Table 2).

### Statistical criteria (cutoff optimization)

Due to the partial overlap in the ultrasound score ranges between the BAMIC grade groups 2A/2B/2C and 3B/3C/4, a performance analysis was conducted to determine the most appropriate cutoff point between these two groups. All possible score values were tested as separation thresholds; sensitivity and specificity were calculated for each to distinguish between groups. The optimum point was defined by the Youden index (sensitivity + specificity − 1), which reached a maximum value of 8 points, confirming the observed distributional boundary (Table 2).

**Table 2:** Correspondence between SOMICS categories and BAMIC grades.

SOMICS category^*^	SOMICS score	BAMIC grade(s)
0	0	0A/0B
1	1–4	1A/1B
2 (2^*^)	5–7	2A/2B/2C
3 (3^*^)	≥ 8	3B/3C/4

* An asterisk added to SOMICS category 2 or 3 (2* or 3*) denotes a higher severity pattern due to a tendon tear, identifying injuries that are more likely to be associated with a delayed return to play.

Grouping BAMIC grades 2A, 2B, and 2C to correspond with SOMICS category 2 and BAMIC grades 3B, 3C, and 4 to correspond with SOMICS category 3 presents a limitation because grades 2C and 3C, for instance, which involve tendinous injury, are associated with longer return-to-play durations compared with grades 2A/2B and 3B, respectively^**(^15^)**^. To address the prognostic heterogeneity in advanced BAMIC grades, particularly those with tendinous involvement, a modifier was added to the SOMICS categories. Cases with sonographic evidence of tendinous tear were further denoted with an asterisk (*), specifically applied to SOMICS categories 2 and 3 when a predominant tendinous tear was present (Supplementary Figure 1).

There was a statistically significant association between the SOMICS categories (0, 1, 2, and 3) and the BAMIC grades (0A/0B; 1A/1B; 2A/2B/2C; 3B/3C/4) according to Pearson’s chi-square test (χ^2^ = 52.53; degrees of freedom = 9; *p* = 3.6 × 10^−8^). Cramer’s *V* coefficient was 0.59 (95% CI: 0.47–0.72), indicating a moderate to strong association between the two classification methods (Table 3).

**Table 3:** Measures of association between SOMICS categories and BAMIC grades.^*^

SOMICS category	BAMIC grade(s)				Total
	0A/0B	1A/1B	2A/2B/2C	3B/3C/4	
	(n)	(n)	(n)	(n)	(n)
0	19	6	1	1	27
1 (1–4)	0	1	0	0	1
2 (5–7)	0	5	6	1	12
3 (≥ 8)	0	2	1	8	11
Total	19	14	8	10	51

* Chi-square test (χ^2^ = 52.53; degrees of freedom = 9; *p* = 3.6 × 10^−8^) and Cramer’s *V* coefficient (*V* = 0.59; 95% CI: 0.47–0.72).

Spearman’s correlation coefficient between the continuous ultrasound score and the BAMIC grades was high (ρ = 0.80; *p* < 0.001; 95% CI: 0.66–0.91), confirming an increasing ordinal trend between the scales (Table 4). The categorical agreement between the four SOMICS categories and the four BAMIC grades was also substantial, with a quadratic weighted kappa of 0.77 (95% CI: 0.59–0.90) and an overall accuracy of 66.7% (95% CI: 52.9–78.4%), as summarized in Table 4. Additional detailed data, including numerical agreement results, sociodemographic characteristics, and illustrative measurements, are provided in the Supplementary Material.

**Table 4:** Association, correlation, and agreement measures between the SOMICS and the BAMIC.

Metric	Estimate	*p*-value	95% CI (bootstrap)
Chi-square (df = 9)	52.53	3.6 × 10^−8^	34.23–79.11
Cramer’s *V*	0.586	-	0.473–0.719
Spearman’s rho	0.800	< 0.001	0.657–0.911
Quadratic weighted kappa	0.770	-	0.594–0.901
Overall accuracy	0.667	-	0.529–0.784

df, degrees of freedom.

## DISCUSSION

The selection of the BAMIC as the reference standard was based on its anatomical framework and its integration into routine clinical practice at our institution, which facilitated methodological alignment with the imaging-driven structure of the SOMICS. The analyses demonstrated high consistency in the ultrasound and MRI readings, with intraobserver and interobserver agreement ranging from substantial to excellent. These results support the reliability of the imaging assessments in this study and indicate that the observed agreement is independent of reader variability.

Two epidemiological characteristics of the sample align with data in the literature: the predominance of hamstring, adductor, and quadriceps injuries; and the high frequency of myotendinous involvement^**(^3^)**^. Although injuries of the obturator externus muscle are relatively uncommon, they were observed in nearly 9% of cases; such injuries are associated with specific soccer-related movements, particularly sliding tackles that involve forced hip abduction^**(^16^,^17^)**^.

In this context, the SOMICS is proposed as a novel ultrasound-based grading system for myotendinous injuries in soccer players. The system is designed to leverage the inherent advantages of ultrasound, such as accessibility, low cost, dynamic assessment, and rapid acquisition, while incorporating severity-oriented imaging features, including power Doppler findings, to identify injuries with greater anatomical disruption.

Several classification systems have been proposed to grade sports-related muscle injuries and estimate the prognosis or risk of recurrence^**(^4^)**^. Early approaches from the 1960s and 1970s relied primarily on clinical presentation and pathophysiological concepts. In the 1980s, the introduction of imaging techniques enabled objective assessment of severity. Paoletta et al.^**(^4^)**^ and Peetrons^**(^11^)**^ emphasized lesion location and the extent of tissue involvement, whereas Lee & Healy^**(^18^)**^ highlighted the importance of morphological and vascular features on Doppler imaging. Later, Chan et al.^**(^19^)**^ introduced the concept of anatomically distinct injuries, including myotendinous, intramuscular, and myofascial lesions. Despite those advances, none of the previously proposed ultrasound-based classifications integrated vascular flow parameters in a structured manner or systematically compared sonographic severity scores with established MRI-based systems such as the BAMIC, Munich Consensus, and Barcelona/Aspetar classifications^**(^10^,^13^,^20^)**^. The SOMICS was developed to address these gaps and add new parameters related to repair tissue formation^**(^4^,^14^–^17^,^21^-^23^)**^, by integrating anatomical location, tissue disruption, extent of involvement, and Doppler findings into a weighted scoring framework in which individual features contribute proportionally to overall severity. This approach produces severity categories that are conceptually analogous to those used in standardized imaging reporting systems such as the Breast Imaging Reporting and Data System^**(^24^)**^, Thyroid Imaging Reporting and Data System^**(^25^)**^, and Lung Computed Tomography Screening Reporting and Data System^**(^26^)**^, all of which prioritize reproducibility, structured communication, and clinical utility over fine-grained prognostic subclassification.

A primary challenge in correlating the SOMICS categories and BAMIC grades was the prognostic heterogeneity observed in higher-grade injuries, particularly those affecting tendinous structures. Previous BAMIC studies have demonstrated that intratendinous injuries (grades 2C and 3C) are associated with significantly longer return-to-play times compared with purely muscular or myotendinous injuries^**(^15^)**^. Because ultrasound consistently enabled visualization of tendon injuries, and to ensure simplicity and reproducibility, the SOMICS was not subdivided into multiple tendon-specific subcategories. Instead, an asterisk (*) modifier is applied to SOMICS categories 2 and 3 when there was sonographic evidence of a tendinous tear, thereby alerting the physician to the presence of a more severe injury.

Several limitations of this study should be acknowledged, including the relatively small convenience sample, uneven distribution across the BAMIC grades, inclusion of comparison groups with different age profiles, and limited prognostic resolution due to grouping tendinous and non-tendinous lesions within the SOMICS categories. In addition, the predominance of adolescent athletes may limit the applicability of the findings to older or non-athletic populations. Despite these limitations, this work introduces an original ultrasound-based classification system that addresses a significant gap in musculoskeletal imaging: the lack of a structured, severity-based framework for grading muscle injuries in adolescents. At this exploratory stage, the primary objective was to demonstrate feasibility, internal consistency, and categorical alignment with an established reference classification under real-world clinical conditions, rather than to establish definitive prognostic equivalence with MRI-based systems. Future studies with larger sample sizes, prospective designs, and external validation in independent cohorts will be essential to refine the SOMICS criteria, assess its prognostic performance, and confirm its applicability across different populations and competitive levels.

In summary, the results demonstrate that the SOMICS shows significant association, ordinal consistency, and substantial agreement with the BAMIC for severity stratification. The SOMICS is not intended to replace MRI-based classifications for prognostic decision-making, particularly in elite athletes, but rather to provide a structured ultrasound-based approach to severity stratification. Within these constraints, the SOMICS may be particularly useful in clinical settings where MRI is not readily available or as a first-line imaging tool.

## Figures and Tables

**Supplementary Table 1:** Sociodemographic characteristics of the athletes evaluated.

Variable	(N = 51)
Sex, n (%)	
Male	51 (100)
Age (years), mean (range)	15.8 (11–19)
Type of sport, n (%)	
Field soccer	47 (92.2)
Field and indoor soccer	4 (7.8)
Position, n (%)	
Goalkeeper	2 (3.9)
Defender	8 (15.7)
Full-back	10 (19.6)
Midfielder	15 (29.4)
Forward	16 (31.4)

**Supplementary Figure 1:**
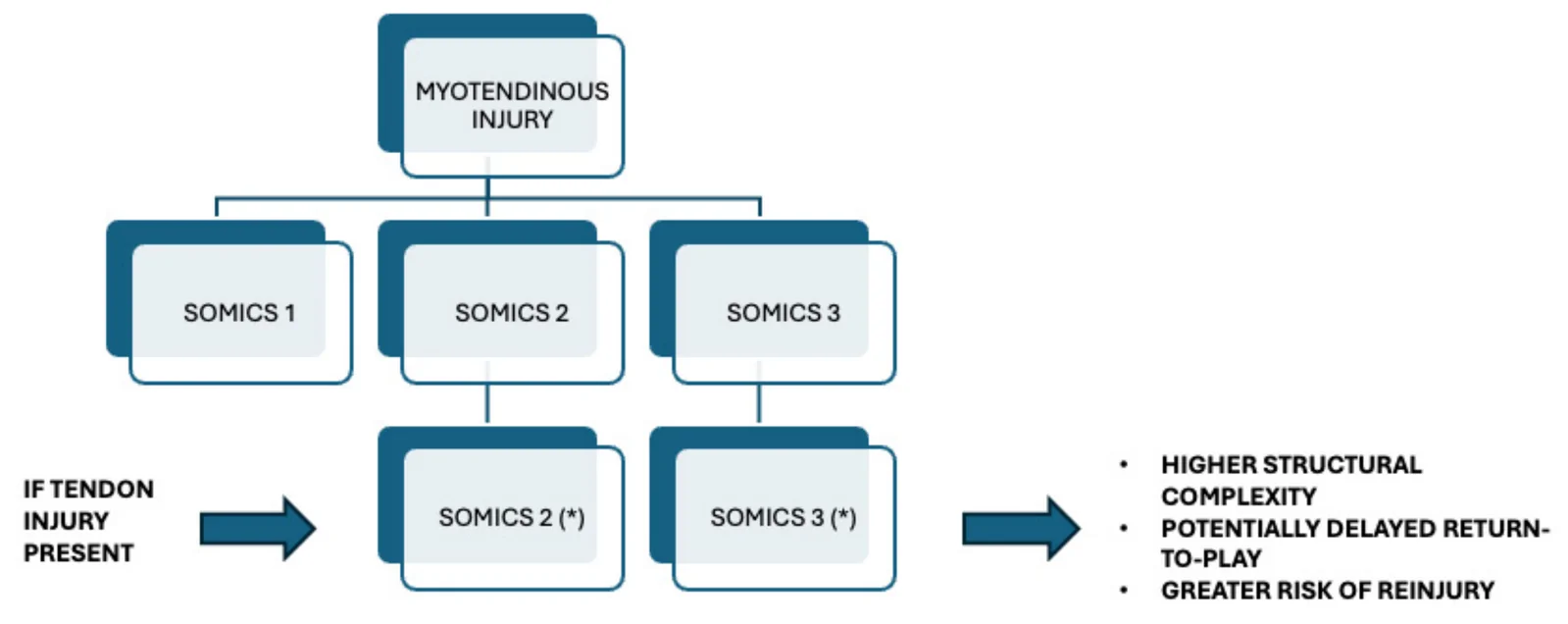
Flow chart of the SOMICS categories and the importance of tendon injuries.

**Supplementary Table 2:** Intraobserver and interobserver agreement for ultrasound and MRI assessments.

Modality	Agreement type	Simple agreement	Kappa	95% CI
		(%)		
Ultrasound	Reader 1	96.1	0.92	0.80–1.00
Ultrasound	Reader 2	94.1	0.86	0.67–1.00
Ultrasound	Interobserver	86.3	0.65	0.43–0.84
MRI	Reader 1	94.1	0.86	0.68–1.00
MRI	Reader 2	98.0	0.96	0.87–1.00
MRI	Interobserver	86.3	0.70	0.49–0.88

**Supplementary Table 3:** Distribution of SOMICS scores among the athletes evaluated (N = 51).

SOMICS score	n (%)
0	27 (52.9)
1	12 (23.5)
2	4 (7.8)
3	11 (21.6)
4	5 (9.8)
5	7 (13.7)
6	8 (15.7)
7	2 (3.9)

**Supplementary Table 4:** Distribution of the BAMIC grades among the athletes evaluated (N = 51).

BAMIC grade	n (%)
0A	17 (33.3)
0B	2 (3.9)
1A	5 (9.8)
1B	9 (17.6)
2A	1 (2.0)
2B	4 (7.8)
2C	3 (5.9)
3B	3 (5.9)
3C	5 (9.8)
4	2 (3.9)

## Data Availability

The research data are available upon request.
